# Dynamic hierarchical multi-scale fusion network with axial MLP for medical image segmentation

**DOI:** 10.1038/s41598-023-32813-z

**Published:** 2023-04-18

**Authors:** Zhikun Cheng, Liejun Wang

**Affiliations:** College of Information Science and Engineering, Xinjiang University, Urumqi, 830046 China

**Keywords:** Cancer, Medical research, Engineering

## Abstract

Medical image segmentation provides various effective methods for accuracy and robustness of organ segmentation, lesion detection, and classification. Medical images have fixed structures, simple semantics, and diverse details, and thus fusing rich multi-scale features can augment segmentation accuracy. Given that the density of diseased tissue may be comparable to that of surrounding normal tissue, both global and local information are critical for segmentation results. Therefore, considering the importance of multi-scale, global, and local information, in this paper, we propose the dynamic hierarchical multi-scale fusion network with axial mlp (multilayer perceptron) (DHMF-MLP), which integrates the proposed hierarchical multi-scale fusion (HMSF) module. Specifically, HMSF not only reduces the loss of detail information by integrating the features of each stage of the encoder, but also has different receptive fields, thereby improving the segmentation results for small lesions and multi-lesion regions. In HMSF, we not only propose the adaptive attention mechanism (ASAM) to adaptively adjust the semantic conflicts arising during the fusion process but also introduce Axial-mlp to improve the global modeling capability of the network. Extensive experiments on public datasets confirm the excellent performance of our proposed DHMF-MLP. In particular, on the BUSI, ISIC 2018, and GlaS datasets, IoU reaches 70.65%, 83.46%, and 87.04%, respectively.

## Introduction

Because medical images are affected by equipment, the partial volume effect, and patient position movement, they inevitably have noise and artifacts. At the same time, the lesion areas are complex and diverse, which all present certain obstacles to the physician's diagnosis. As a result, the efficiency and accuracy of diagnosis have increased as doctors are assisted by computers.

With the development of deep learning, the emergence of convolutional neural networks (CNNs)^1^ has played a huge role in the development of medical image segmentation. CNNs perform well in many segmentation tasks, such as multi-organ segmentation through abdominal CT images^2–4^, lesion detection^5–7^, cell segmentation^8–10^, heart segmentation^11–13^, etc. Unfortunately, for the segmentation of high-level networks, the feature maps contain less detail information due to their low resolution. For the low-level networks of segmentation, the feature maps have more noise. The low-level networks also have the characteristics of a small receptive field and weak semantic information representation abilities. However, both high-level semantic information and low-level features are extremely important to the final segmentation result. Effective multi-scale feature fusion contributes to identifying network segment targets more accurately, which is an important way to improve segmentation performance. In order to guide the segmentation of small lesions and multi-lesion regions and increase prediction accuracy, many CNNs have been proposed that fuse low-level features with high-level semantic information. For example, the pure convolutional network U-Net^14^ fuses low-level features into the up-sampling through skip connections. U-Net^14^ has become the baseline for most medical image segmentation tasks and has inspired a large number of researchers to think about U-shaped semantic segmentation networks. V-Net^15^, which is used for 3D image segmentation, also uses skip connections to transmit low-level features. However, these simple skip connections do not achieve cross-scale interaction. Later, it is proposed that U-Net++^16^ indirectly fuses features of several different levels through short skip connections and up-down sampling. MDU-Net^17^ extracts rich semantic information through multi-scale dense connection encoders, decoders, and skip connections. With the deepening of the network, the features of the deep network are greatly offset from the features of the shallow network, and direct feature fusion will lead to semantic conflicts. These conflicts inhibit the learning of detail information, which is not conducive to the establishment of context information for multi-scale features and has negative impacts on segmentation results.

For the reasons outlined above, many researchers have proposed a variety of attention mechanisms to make networks focus on features of greater interest. SE-Net^18^ and Coordinate Attention^19^ use the generated weight sequence to explicitly build the dependency relationship between channels, so as to increase the sensitivity of the model to channel information and make channel information contribute more to the final decision. CBAM^20^ further combines channel attention with spatial attention and has better performance. However, these networks ignore the different proportions of foreground and background information for each feature map at different sampling stages.

Based on the above analysis, we propose dynamic hierarchical multi-scale fusion network with axial mlp (DHMF-MLP) for medical image segmentation, in which we integrate the hierarchical multi-scale fusion (HMSF) module. We generate features with rich semantic and spatial information by fusing features from each stage of the encoder several times. To alleviate semantic conflicts in multi-scale feature fusion and enhance the ability to model the network globally, we propose dynamic spatial linear attention module (DSLA) as a component of HMSF. DSLA includes two parts: the adaptive spatial attention mechanism (ASAM) and the global branching in multi- gated MLP^21^ (Axial-mlp^21^). In the ASAM module, the semantic conflicts between multi-scale features can be adjusted adaptively by learning parameters, and the noise inhibiting segmentation performance can be filtered out to enhance the attention of important features. Axial-mlp^21^ addresses the baseline's (UNeXt^22^) shortcoming in global information modeling with linear computational complexity.

The contribution of this paper can be summarized as follows:

1. We design the HMSF module, which achieves cross-level information interaction. HMSF not only improves the segmentation accuracy of small lesions and multi-lesion areas but also reduces the loss of information caused by pooling structures, fully improving the lack of up-sampling information.
2. We propose the DSLA module and apply it to the HMSF module. One part of the DSLA is the ASAM module, which adaptively adjusts the semantic conflict of multi-scale features with learnable parameters, filters out background noise that inhibits detail learning, and highlights foreground information appropriately. Another part of the DSLA is Axial-mlp^21^, which enhances the global modeling capabilities of the network with less computation.
3. We achieve interaction between different layers, enriching the semantic information and reducing the conflict of fusing different features when compared to UNeXt^22^. Further advancements in global modeling capabilities allow for even better network segmentation performance.
4. The effectiveness of our proposed network has been proven by experiments on three datasets. Compared with other networks, our network is highly competitive.

The remainder of this paper is organized as follows: “Related work” section shows the related work. “Method” section describes our proposed method in detail. “Experiments and analysis” section shows the experiments and analysis. “Conclusion” section gives the conclusion.

## Related work

### Based on the traditional image fusion methods

Spatial domain, transform domain, and their combination make up traditional medical image fusion algorithms. Principal component analysis^23^ is a common fusion technique for medical imaging based on the spatial domain. Nevertheless, spectral and spatial distortion of the merged images are produced by spatial domain approaches. Researchers have moved their attention to the transform domain in an effort to improve the results of fusion. The contour transform^24^, discrete wavelet transform^25^, and pyramid transform^26^ are common examples. Although the transform domain-based approaches produce noise during the fusion process, they have the advantages of excellent structure and distortion avoidance. Better fusion results are obtained when the two procedures are combined. However, based on the traditional fusion methods, on the one hand, they are compelled to employ the same transform for various source images to extract features in order to guarantee the viability of subsequent feature fusion. The fact that this process disregards the variations in the source images' characteristics could result in a subpar representation of the extracted features. On the other hand, the performance of the conventional feature fusion technique is insufficient and too coarse. The technique for integrating deep learning into image fusion gets over these drawbacks of conventional approaches.

### CNN-based methods

The emergence of CNNs has led to rapid development in the field of image segmentation. FCN^27^ is the pioneer of CNNs for image segmentation, opening up a new era of encoder–decoder structure for image segmentation. Subsequently, U-Net^14^ combines encoder features from different levels to reduce information loss from pooling structures, achieve more accurate pixel boundary localization, and generate a plethora of efficient U-shaped segmentation network architectures^28,29^. Some researchers have further improved the structure of CNN-based networks, like Dilated Convolution^30,31^, RefineNet^32^, and PSPNet^33^.These networks are widely used in the field of image segmentation. However, due to the inherent characteristics of convolution, it lacks the ability to perform global context modeling.

### Attention mechanisms

The attention mechanism is designed to focus the network on more important features. Channel attention is weighted by channel direction to automatically obtain the contribution of each channel to the segmentation result. The representative networks are SE-Net^18^, ECANet^34^, and FcaNet^35^. The spatial attention mechanism is weighted along the spatial dimension so that the network can weaken background noise and pay more attention to the foreground information. For example, GE-Net^36^, RA-Net^37^, and SPA-Net^38^ make full use of spatial context information to make the network more efficient in mining regions of interest. However, these attention mechanisms do not take into account the dynamic relationship between foreground and background information for different scale features. Self-attention is a variant of the attention mechanism that aims to reduce dependence on external information and to use information inherent within features for the interaction of attention whenever possible. Like non-local^39^, the global context is modeled to effectively capture feature dependencies over long distances. OCR-Net^40^ models from a category perspective, which uses the results of coarse segmentation as the object of modeling and finally assigns weights to each query point. The bad news is that these calculations are relatively large.

### MLP-based methods

MLP-Mixer^41^ uses multilayer perceptron (MLP) to replace the convolution operation of CNN and the self-attention mechanism in Transformer^42–44^. MLP-Mixer^41^ builds contextual and inter-channel correlations between tokens through cross-position and per-position operations, respectively. gMLP^45^ achieves better performance than MLP-Mixer^40^ with fewer parameters and simpler gating mechanism. AS-MLP^46^ aligns different features to the same channel by parallel axial shift operations, and the full connection along the channel position achieves a cross-shaped field of view. AS-MLP^46^ is the first MLP architecture to migrate to downstream tasks. The above network does not balance model performance and computational redundancy. MAXIM^21^ employs multi-axis gated MLP to extract local and global information simultaneously, with cross-gated to achieve information interaction.

Compared to the above network, each layer of features in the DHMF-MLP encoder section interacts with its upper layer of features, reducing deviations between features. DHMF-MLP can not only adaptively adjust semantic conflicts in multi-scale feature fusion according to image properties but also extract perfect global context information.

## Method

This section describes our proposed DHMF-MLP network as well as the research motivation. We will briefly introduce the overall architecture of the network in “Overall network structure”. We introduce the principle of the HMSF module in “Hierarchical multi-scale fusion module (HMSF)”. We elaborate on the principle of the DSLA module in "Dynamic spatial linear attention module (DSLA)".

### Research motivation

As down-sampling proceeds, image information is lost and feature offsets can occur. By fusing encoder features layer by layer, interaction between higher-level features and their relative lower-level features can be achieved, and bias between features can be reduced. Semantic conflicts arise during the fusion process, and by adaptively adjusting the conflicts, consistent multi-scale feature sequences can be generated, facilitating the learning of important features. The fused features contain rich semantic information and are up-sampled from the decoder's bottom, reducing the semantic gap in the skip connections part and improving prediction accuracy. Furthermore, human tissues are highly similar, and both global and local information are critical. While UNeXt^22^ performs well in local feature extraction, it underperforms in global contextual information. We cite the Axial-mlp^21^ to improve the overall network global modelling capability. Therefore, we propose the DHMF-MLP with the above issues fully in mind.

### Overall network structure

**Figure 1:**
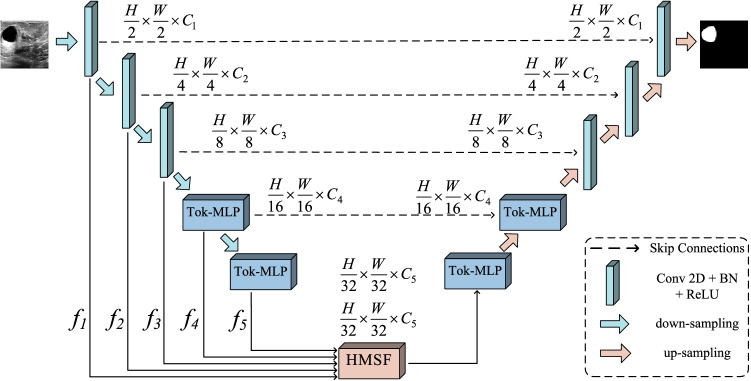
Overall architecture of the DHMF-MLP (created by ‘Microsoft Office Visio 2013’ URL: https://www.microsoft.com/zh-cn/microsoft-365/previous-versions/microsoft-vision-2013).

The overall architecture of DHMF-MLP is shown in Fig. 1. The network is U-shaped structure consisting of encoder–decoder and skip connections between the encoder–decoder. In the encoder part, low-level features and high-level features with marvelous local characteristics are gradually extracted by convolution and Tok-MLP. Intermediate features are retained to form multi-scale feature sequence {${f}_{1},{f}_{2},{f}_{3},{f}_{4},{f}_{5}$}, which is used for skip connections and input of HMSF. To mitigate the simple semantic properties of medical images, multi-scale feature sequence is fed into the HMSF module. In this part, features containing richly detail information and high-level semantic information can be generated for use as input for up-sampling. In the decoder part, the generated multi-scale feature is up-sampled by bilinear interpolation and passed through Tok-MLP and convolution to obtain the final prediction map. For all experiments in the DHMF-MLP network, we set C1, C2, C3, C4 and C5 to 32, 64, 128, 160 and 256, respectively.

### Tok-MLP

The channel is divided into h parts for the input feature T, then axially shifted along the w-dimension and *Tokenized* to obtain ${T}_{W}$. The formula (1) is as follows:

1 $${T}_{W}=Tokenized\left({shift}_{w}\left(\uprho \left(T\right)\right)\right),$$

where *ρ* and ${shift}_{w}$ indicates the division along the channel dimension and shifted along the w-dimension, respectively.

${T}_{W}$ performs *MLP* along the channel dimension to map the number of channels into 768 dimensions, followed by 3 × 3 *DWConv* and *GLEU* to obtain ${T}_{1}$. The formula (2) is as follows:

2 $${T}_{1}=GELU\left(DWConv\left(MLP\left({T}_{W}\right)\right)\right),$$

where $DWConv$ indicates 3 × 3 depth-wise convolution.

${T}_{1}$ is similarly shifted along the H-dimension to obtain ${\mathrm{T}}_{H}$. The module output is obtained by concatenating the residuals after mapping ${\mathrm{T}}_{H}$ into the original input feature dimension. By generating a random window, the module extracts excellent local features. The formula (3) and (4) is as follows:

3 $${\mathrm{T}}_{H}= MLP(Tokenized\left({shift}_{h}\left(\uprho \left(T\right)\right)\right)),$$

4 $$\mathrm{output}=\mathrm{ T }\oplus \mathrm{ FC}\left({\mathrm{T}}_{H}\right),$$

where FC and ${shift}_{h}$ indicates fully connected layers and shifted along the h-dimension, respectively. $\oplus$ denotes element-wise addition.

### Hierarchical multi-scale fusion module (HMSF)

It is well known that the low-level features of the segmentation network contain more fine-grained information, which is helpful for the segmentation of small lesions. The deep segmentation network is able to extract more high-level semantic information, which can improve the accuracy of segmentation. Moreover, the rich multi-scale information, which fuses features with different receptive fields, facilitates the segmentation of multi-lesion regions.

**Figure 2:**
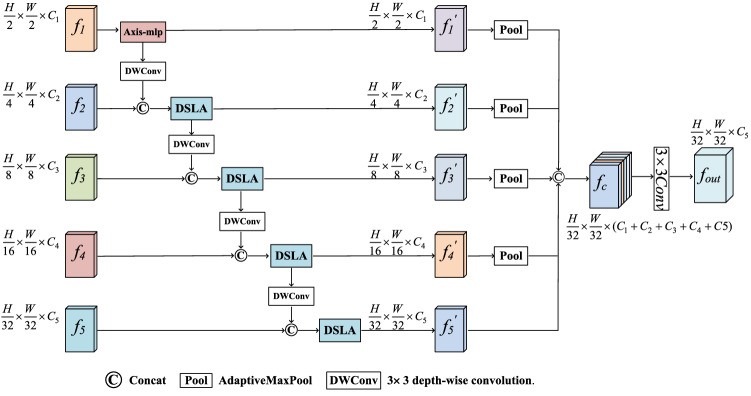
Overall architecture of HMSF module (created by ‘Microsoft Office Visio 2013’ URL: https://www.microsoft.com/zh-cn/microsoft-365/previous-versions/microsoft-vision-2013).

In this paper, we propose the HMSF module. The structure of the HMSF module is shown in Fig. 2. There are two fusions of HMSF. The first, features from each encoder stage are fused with relative low-level features. The second, the result obtained after the first fusion is fused again, and the fused result is used as the input for up-sampling.

Formally, the HMSF module has five input scales ${f}_{i}$ (i = 1, 2, 3, 4, 5). For ${f}_{1}$, there are no relatively low-level features, so no feature fusion or semantic conflict is required for adjustment. Only Axial-mlp^21^ is performed to create global context to obtain ${f}_{1}^{{\prime}}$*,* the formula (5) is as follows:

5 $${f}_{1}^{{\prime}}=MLP\left({f}_{1}\right),$$

where *MLP* represents Axial-mlp^21^. It is a branch of the DSLA, as discussed in detail in "Dynamic spatial linear attention module (DSLA)".

For feature ${f}_{i}$ (i = 2, 3, 4, 5), its relative low-level feature ${f}_{i-1}^{{\prime}}$ (i = 2, 3, 4, 5) is down-sampled by 3 × 3 $DWConv$ to the resolution of ${f}_{i}$(i = 2, 3, 4, 5). The feature obtained from down-sampling is concatenated with ${f}_{i}$ to retain more channel information. *DSLA* module is applied to obtain new fusion feature ${f}_{i}^{{\prime}}$ (i = 2, 3, 4, 5). Reserve the intermediate value of this feature, which serves as input for the next stage of fusion. In this way, we can generate consistent multi-scale sequences {${f}_{1}^{{\prime}},{f}_{2}^{{\prime}},{f}_{3}^{{\prime}},{f}_{4}^{{\prime}},{f}_{5}^{{\prime}}$} with rich detail and high-level semantic information. The formula (6) is as follows:

6 $${f}_{i}^{{\prime}}=DSLA(Concat({DWConv}_{3\times 3}\left({f}_{i-1}^{{\prime}}\right), {f}_{i}^{{\prime}}), (\mathrm{i}=\mathrm{2,3},\mathrm{4,5}),$$

where *Concat* is the concatenation operation. We adapt ${DWConv}_{3\times 3}$ to represent 3 × 3 depth-wise convolution.

Then {${f}_{1}^{{\prime}},{f}_{2}^{{\prime}},{f}_{3}^{{\prime}},{f}_{4}^{{\prime}}$} are down-sampled to the size of ${f}_{5}^{{\prime}}$ by adaptiveMaxpool. The features after down-sampling are concatenate together along the channel dimensions, and then $3\times 3$ convolution is carried out to obtain the final output ${f}_{out}$.The ${f}_{out}$ can be obtained by the following formula (7):

7 $${f}_{out}= {Conv}_{3\times 3}(Concat(pool\left({f}_{i}^{{\prime}}\right), (\mathrm{i}=\mathrm{1,2},\mathrm{3,4})),(\mathrm{i}=\mathrm{1,2},\mathrm{3,4},5))),$$

where ${f}_{i}^{{\prime}}$ denotes the output of the i-layer encoder in the first fusion process.${f}_{out}$ is the final fusion output of the HMSF module , *pool* we use AdaptiveMaxPool.

### Dynamic spatial linear attention module (DSLA)

**Figure 3:**
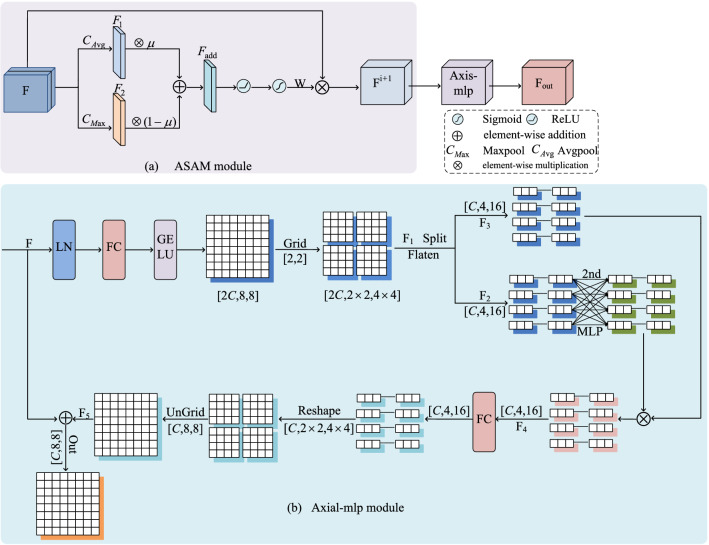
Overall architecture of DSLA module (created by ‘Microsoft Office Visio 2013’ URL: https://www.microsoft.com/zh-cn/microsoft-365/previous-versions/microsoft-vision-2013).

As down-sampling proceeds, there exists positional deviation between low-level features and high-level features. To resolve the semantic conflicts that occur when they are fused and to enhance the global modeling capability of the network, this paper proposes the DSLA module. As shown in Fig. 3, the DSLA consists of two parts. On the one hand, ASAM is used for feature selection. On the other hand, Axial-mlp^21^ is used to enhance the global contextual information of the fused features.

#### Adaptive spatial attention mechanism (ASAM)

Inspired by SE-Net^18^, we propose an efficient mechanism, which is shown in Fig. 3a. In this part, we conduct Avgpool and Maxpool of input ${F}^{\mathrm{B}\times \mathrm{C}\times \mathrm{H}\times \mathrm{W}}$ features along channel dimensions to obtain corresponding feature maps ${F}_{1}^{B\times 1\times H\times W}$,${F}_{2}^{B\times 1\times H\times W}$. In order to adaptively adjust the dynamic balance between redundant background information and foreground information according to the characteristics of the different scale features, we apply the learnable parameters μ(0 < μ < 1), 1 − μ multiplied by the Avgpool and Maxpool, respectively. After learning the two features are summed to get ${F}_{add}^{B\times 1\times H\times W}$. ${F}_{add}^{B\times 1\times H\times W}$ and sigmoid are operated to obtain the adaptive weight parameter ${w}^{B\times 1\times H\times W}$, which is used for feature selection. The ASAM module is calculated by the following formula (8):

8 $${{F}^{i+1}= \sigma (ReLU(\mu \otimes C}_{Avg}\left(F\right) \oplus (1-\mu )\otimes {C}_{Max}(F))) \otimes F,$$

where ${F}^{i+1}$ represents the output after feature selection, ${C}_{Avg}$ is the spatial Avgpool of features compressed into individual channel along the spatial direction of channel dimension, ${C}_{Max}$ is the spatial Maxpool of features compressed into a individual channel along the spatial direction of channels dimension. $\otimes$ denotes element-wise multiplication, and $\oplus$ denotes element-wise addition. $\sigma$ is the sigmoid function.

#### Axial-mlp^21^

In order to enhance the ability of the network to capture global context information and reduce computational complexity, Axial-mlp^21^ is constructed by processing non-overlapping image patches of fixed size to achieve this goal. The structure of Axial-mlp^21^ is shown in Fig. 3b. For the input feature ${F}^{\mathrm{C\times H\times W}}$, the channel is mapped to 2C, and then the new feature is gridded into the shape ${{F}_{1}}^{2\mathrm{C}\times (\mathrm{d}\times \mathrm{d})\times (\frac{H}{d}\times \frac{W}{d})}$. We set the size of the grid to be fixed $(\mathrm{d}\times \mathrm{d})$. In this paper, we set d = 8. The formula (9) is as follows:

9 $${F}_{1}=\updelta (\upsigma \left(FC\left(LN\left(F\right)\right)\right))$$

where *LN*, *FC* represents LayerNorm and fully connected layers, respectively. σ denotes GELU. δ denotes grid operation.

After encapsulation, the channel dimension is divided into two branches to obtain ${{F}_{2}}^{\mathrm{C}\times (\mathrm{d}\times \mathrm{d})\times (\frac{H}{d}\times \frac{W}{d})}$, ${{F}_{3}}^{\mathrm{C}\times (\mathrm{d}\times \mathrm{d})\times (\frac{H}{d}\times \frac{W}{d})}$. ${{F}_{2}}^{\mathrm{C}\times (\mathrm{d}\times \mathrm{d})\times (\frac{H}{d}\times \frac{W}{d})}$ performs MLP in the second dimension. The result of ${{F}_{2}}^{\mathrm{C}\times (\mathrm{d}\times \mathrm{d})\times (\frac{H}{d}\times \frac{W}{d})}$ performing MLP is fused with ${{F}_{3}}^{\mathrm{C}\times (\mathrm{d}\times \mathrm{d})\times (\frac{H}{d}\times \frac{W}{d})}$ via multiplicative gating to obtain ${{F}_{4}}^{\mathrm{C}\times (\mathrm{d}\times \mathrm{d})\times (\frac{H}{d}\times \frac{W}{d})}$. The formula (10) is as follows:

10 $${F}_{4}=\mathrm{MLP}({F}_{2}) \otimes {F}_{3},$$

where $\otimes$ denotes element-wise multiplication.

The output of the multiplication gate performs reshape and grid reassembly operations to obtain ${{F}_{5}}^{\mathrm{C\times H\times W}}$.Finally, the output of the Axial-mlp^21^ is obtained by adding ${F}^{\mathrm{C\times H\times W}}$ to ${{F}_{5}}^{\mathrm{C\times H\times W}}$. The out of the Axial-mlp^21^ module is calculated by the following formula (11), (12):

11 $${F}_{5}=\mathrm{\varphi }(\mathrm{FC}\left({F}_{4}\right)),$$

12 $$\mathrm{out}={F}_{5} \oplus \mathrm{ F},$$

where FC represents fully connected layers. $\mathrm{\varphi }$ denotes reshape and ungrid operation. $\oplus$ denotes element-wise addition.

## Experiments and analysis

### Datasets

(1) Breast UltraSound Images (BUSI)^47^: ultrasound images and corresponding segmentation images of normal, benign, and malignant breast cancer cases were collected. We use only benign and malignant images (647 images) and resize all images to 256 × 256. (2) International Skin Imaging Collaboration (ISIC 2018)^48^: the dataset consists of skin images containing cases and corresponding segmentation images of skin lesions, including a total of 2594 images. We resize all images to 512 × 512. (3) GlaS^49^: the dataset consists of 165 microscopic images of hematoxylin and eosin-stained slides, all of which are resized to 256 × 256.

### Implementation details

We utilize the Pytorch framework to develop DHMF-MLP. Consistent with the UNeXt^22^ loss function scaling, we adopt a combination of binary cross entropy (BCE) and dice loss (Dice) for training. The total loss L between prediction ${{\hat{\text{y}}}}$ and target y is expressed as:

13 $${\text{L}} = 0.5{\text{BCE}}\left( {{\hat{\text{y}}},{\text{y}}} \right) + {\text{Dice}}\left( {{\hat{\text{y}}},{\text{y}}} \right)$$

We use Adam optimizer to train the model with the initial learning rate of $1{e}^{-4}$ and momentum of 0.9. The training times are 400 epochs. Eight batches of training are used on the BUSI and ISIC 2018 datasets, and four batches are used on the GlaS datasets. The rotation and flipping techniques are adopted as data augmentation methods to force the model to learn more robust features, so as to effectively improve the generalization ability of the model. We randomly divide all datasets by 8:2 for training and testing, respectively. We evaluate our method on three datasets using IoU, Dice, Sensitivity (SE), Accuracy (Acc), Presion (PPV), and Specificity (SP). All our training is done on a Tesla V100-PCIE GPU.

### Evaluation metrics

We exploit the IoU, Dice, SE, Acc, PPV, and SP segmentation metrics to quantify the segmentation ability of DHMF-MLP. For instance, IoU is used to assess the degree of similarity between prediction and ground truth. SE is a measure of the ability to correctly identify pixels that are not in the region of interest in a segmentation experiment. The formula is shown below:

14 $$\mathrm{Iou}= \frac{\mathrm{TP}}{\mathrm{FP}+\mathrm{TP}+\mathrm{FN}} ,$$

15 $$\mathrm{Dice}= \frac{2\mathrm{TP}}{2\mathrm{TP}+\mathrm{FP}+\mathrm{FN}} ,$$

16 $$\mathrm{SE}= \frac{\mathrm{TP}}{\mathrm{TP}+\mathrm{FN}} ,$$

17 $$\mathrm{Acc}= \frac{\mathrm{TP}+\mathrm{TN}}{\mathrm{TP}+\mathrm{FP}+\mathrm{FN}+\mathrm{TN}} ,$$

18 $$\mathrm{PPV}= \frac{\mathrm{TP}}{\mathrm{TP}+\mathrm{FP }} ,$$

19 $$\mathrm{SP}= \frac{\mathrm{TN}}{\mathrm{TN}+\mathrm{FP}} ,$$

where TP denotes that the sample is deemed positive and is, in fact, positive. TN denotes that the sample has been judged to be negative and is, in fact, negative. FP denotes that the sample is thought to be positive but is actually negative. FN denotes that the sample is thought to be negative but is actually positive.

### Training process

**Figure 4:**
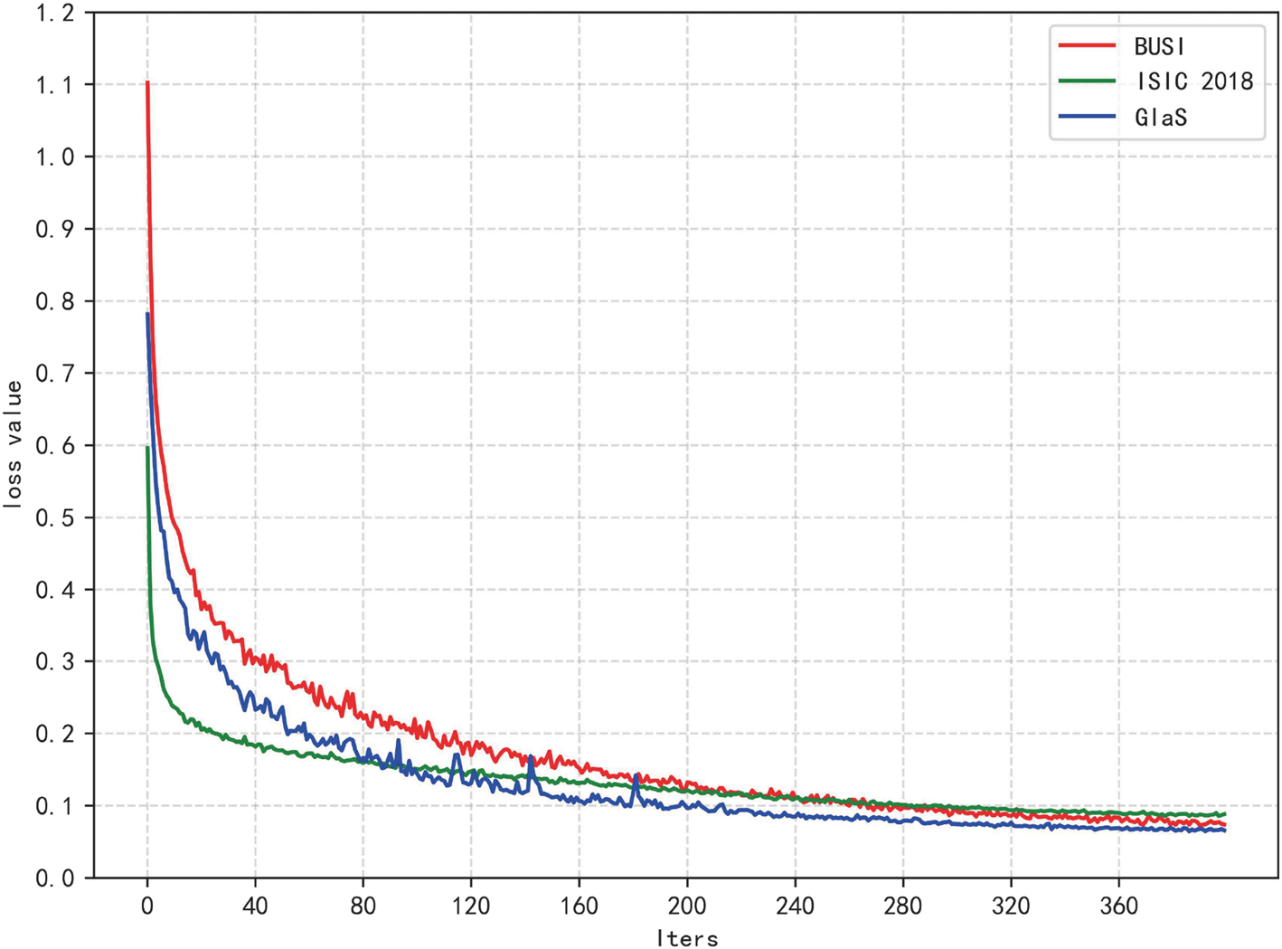
Training loss variation curves for different datasets.

Figure 4 shows a relatively "perfect" loss curve. At the beginning of the training phase, the loss value decreases significantly, indicating a suitable learning rate and a gradient descent process. After a certain stage of learning, the loss curve plateaus.

### Comparative experiment

In order to further measure the effectiveness of the proposed DHMF-MLP network for lesion segmentation, we conduct comparative tests on the BUSI, ISIC 2018, and GlaS datasets. The network architectures used in our comparative experiments include the most advanced CNN-based networks, such as U-Net^14^, U-Net++ ^16^, U-Net3+ ^28^, Att-Unet^29^, and transformer-based network architectures, such as TransUnet^42^ and MedT^50^. We also make comparisons with UNeXt^22^, the network based on MLP. In the following, we will conduct the quantitative and qualitative analysis of the comparative test results. Moreover, the number of Parameters in each network is maintained to two decimal places.

#### Evaluation of the BUSI dataset

##### Quantitative result analysis

The quantitative comparison results of the BUSI dataset on different methods are depicted in Table 1. Based on the traditional convolution methods, they still have good performances. U-Net3+ ^28^ even outperforms the MedT^50^ network based on the transformer method, with the best PPV and SP. However, there is a big gap between the overall performance of the CNN-based methods and DHMF-MLP. IoU, Dice, SE, and ACC are 8.14%, 5.76%, 10.95%, and 0.59% higher than U-Net3+ ^28^, respectively. We note that the 4.54 M parameters of DHMF-MLP are also relatively low compared to the 26.97 M parameters of U-Net3+ ^28^. It shows that DHMF-MLP is efficient in its segmentation performance.

**Table 1:** Comparison results of the proposed method on BUSI dataset.

Method	Year	Params (in M)	IoU	Dice	SE	ACC	PPV	SP
U-Net^14^	2015	34.53	63.98	76.52	73.36	95.77	81.23	98.24
U-Net++ ^16^	2015	9.16	64.09	76.21	73.49	95.74	80.74	98.31
U-Net3+ ^28^	2020	26.97	65.33	77.73	73.30	96.02	**84.36**	**98.64**
TransUnet^42^	2021	105.32	66.59	79.60	79.83	95.99	79.76	97.80
MedT^50^	2021	1.56	62.20	75.85	74.76	95.34	78.56	97.77
UNeXt^22^	2022	2.52	67.44	79.55	77.11	96.31	83.29	98.42
(Ours)	2022	4.54	**70.65**	**82.21**	**81.33**	**96.59**	83.90	98.28

Significant values are in [bold].

##### Qualitative result analysis

**Figure 5:**
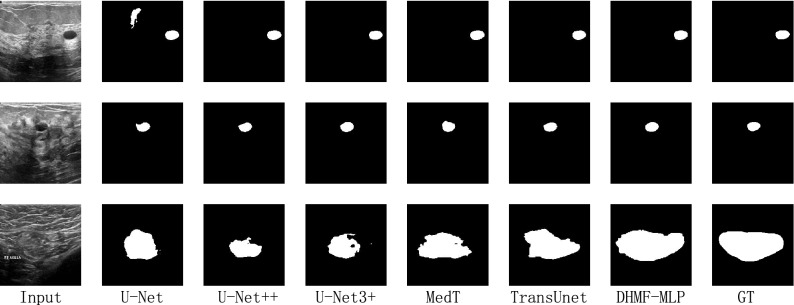
Qualitative comparison results for the BUSI dataset. From left to right: Input, U-Net^14^, U-Net++ ^16^, U-Net3+ ^28^, MedT^50^, TransUnet^42^, DHMF-MLP and GT. (created by ‘Microsoft Office Visio 2013’ URL: https://www.microsoft.com/zh-cn/microsoft-365/previous-versions/microsoft-vision-2013).

The qualitative comparison results of the BUSI dataset using different methods are presented in Fig. 5. According to the third row of Fig. 5, due to the inherent local characteristics of traditional convolution, the control ability of global modeling is insufficient, resulting in under-segmentation. In contrast to the methods based on transformers, we not only achieve cross-scale interaction but also adaptively adjust the semantic conflicts that arise during fusion according to image characteristics. As can be seen in Fig. 5, the DHMF-MLP segmentation is more accurate and complete. From the qualitative analysis, the validity of the HMSF module is verified.

#### Evaluation of the ISIC 2018 dataset

##### Quantitative result analysis

Results of the quantitative comparison of the ISIC 2018 dataset on different methods according to Table 2, DHMF-MLP has all the best segmentation metrics. Among the other baseline models, the TransUnet^42^, based on the Transformer method, has the best PPV and SP. However, the number of DHMF-MLP parameters is very low compared to TransUnet^42^, which is also a relatively lightweight model. These experiments have verified the consistency of the foregoing.

**Table 2:** Comparison results of the proposed method on ISIC 2018 dataset.

Method	Year	Params (in M)	IoU	Dice	SE	ACC	PPV	SP
U-Net^14^	2015	34.53	73.02	83.73	81.90	93.46	87.55	97.21
U-Net++ ^16^	2015	9.16	74.48	84.80	85.25	93.76	86.32	96.70
U-Net3+ ^28^	2020	26.97	78.66	87.81	85.91	94.92	90.65	97.74
TransUnet^42^	2021	105.32	80.69	89.08	86.81	95.45	92.19	97.96
MedT^50^	2021	1.56	79.02	88.00	86.67	95.13	90.34	97.52
UNeXt^22^	2022	2.52	82.55	90.28	89.08	95.87	92.14	97.91
(Ours)	2022	4.54	**83.46**	**90.84**	**89.69**	**96.18**	**92.54**	**98.08**

Significant values are in [bold].

##### Qualitative result analysis

**Figure 6:**
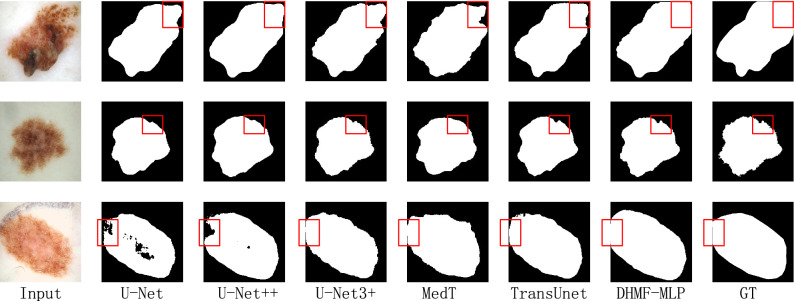
Qualitative comparison results for the ISIC 2018 dataset. From left to right: input, U-Net^14^, U-Net++ ^16^, U-Net3+ ^28^, MedT^50^, TransUnet^42^, DHMF-MLP and GT. (created by ‘Microsoft Office Visio 2013’ URL: https://www.microsoft.com/zh-cn/microsoft-365/previous-versions/microsoft-vision-2013).

Figure 6 provides exemplary qualitative results generated by different methods for several challenging cases from the ISIC 2018 dataset. According to the qualitative analysis results of the red box position in the first row, it is obtained that DHMF-MLP effectively measures the relationship between background information and foreground information and improves the segment effect. Because of the simple semantics of medical images, rich multi-scale information is beneficial for improving segmentation accuracy. Combined with the importance of global context information to the segmentation performance, DHMF-MLP effectively reduces false negatives and better preserves boundaries compared with other methods. The second and third rows of red box positions in Fig. 6 confirm this view.

#### Evaluation of the GlaS dataset

##### Quantitative result analysis

Table 3 shows the results of the quantitative comparison of the GlaS dataset on different methods. This dataset is characterized by inconsistencies in shape and size and by numerous small lesion areas. Both local feature extraction and global context feature extraction are extremely important for segmentation results. As can be seen in Table 3, TransUnet^42^ improves its performance by using CNN and Transformer to extract local and global contextual information, respectively. UNeXt^22^ achieves great competitive advantages by extracting excellent local features through CNN and shifted MLP. DHMF-MLP considers both local features and global feature extraction. Further better segmentation results from the medical image's own characteristics. Our proposed network (DHMF-MLP) has the best IoU, Dice, ACC, PPV and SP, which is 3.87%, 2.10%, 2.10%, 3.42%, 3.46% higher than UNeXt^22^. It should be noted that the proposed DHMF-MLP is also a relatively lightweight model, which is more feasible in clinical scenarios. There are huge advantages to these advanced methods.

**Table 3:** Comparison results of the proposed method on GlaS dataset.

Method	Year	Params (in M)	IoU	Dice	SE	ACC	PPV	SP
U-Net^14^	2015	34.53	66.18	79.26	**96.67**	75.16	67.79	55.37
U-Net++ ^16^	2015	9.16	68.43	80.83	74.68	83.59	89.90	91.86
Att-UNet^29^	2019	19.17	70.62	82.68	82.97	83.25	83.01	83.61
TransUnet^42^	2021	105.32	80.11	88.85	87.74	89.45	90.23	91.12
MedT^50^	2021	1.56	68.44	80.98	92.19	78.81	73.07	66.32
UNeXt^22^	2022	2.52	83.80	91.12	92.19	91.42	90.16	90.50
(Ours)	2022	4.54	**87.04**	**93.03**	92.85	**93.35**	**93.24**	**93.63**

Significant values are in [bold].

##### Qualitative result analysis

**Figure 7:**
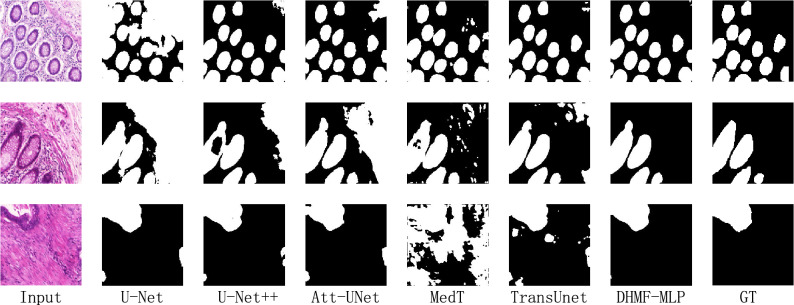
Qualitative comparison results for the GlaS dataset. From left to right: input, U-Net^14^, U-Net++ ^16^, Att-Unet^29^, MedT^50^, TransUnet^42^, DHMF-MLP and GT. (created by ‘Microsoft Office Visio 2013’ URL: https://www.microsoft.com/zh-cn/microsoft-365/previous-versions/microsoft-vision-2013).

Based on the above analysis of the characteristics of the GlaS dataset and the results of the qualitative analysis of the GlaS dataset in the first row of results in Fig. 7, U-Net++ ^16^ and DHMF-MLP enable cross-scale interaction to reduce redundant information interference compared to U-Net^14^. From the second row of results, TransUnet^42^ and MedT^49^ combine the CNN with the Transformer and give better segmentation results of the junction of the lesion area compared to U-Net++ ^16^. DHMF-MLP further considers the feature conflict during fusion compared to TransUnet^42^ and proposes ASAM. As shown in Fig. 7, our method effectively measures foreground and background information. Compared with other methods, verify the feasibility of DHMF-MLP for segmentation.

### Analytical study

**Figure 8:**
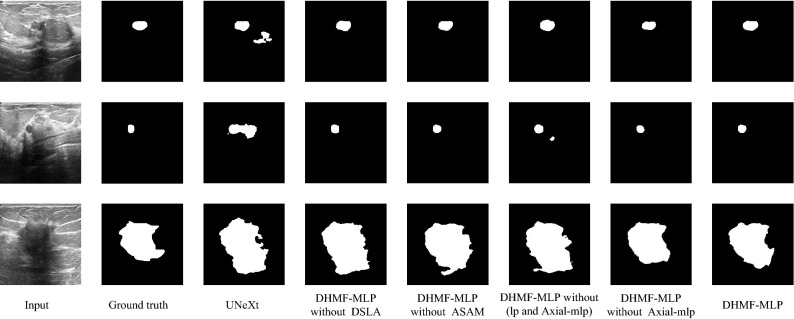
Ablation segmentation results of HMSF block on the BUSI dataset. From left to right: input, ground truth, UNeXt^22^, DHMF-MLP without DSLA, DHMF-MLP without ASAM, DHMF-MLP without (lp and Axial-mlp^21^), DHMF-MLP without Axial-mlp^21^, DHMF-MLP(Ours). (created by ‘Microsoft Office Visio 2013’ URL: https://www.microsoft.com/zh-cn/microsoft-365/previous-versions/microsoft-vision-2013).

**Figure 9:**
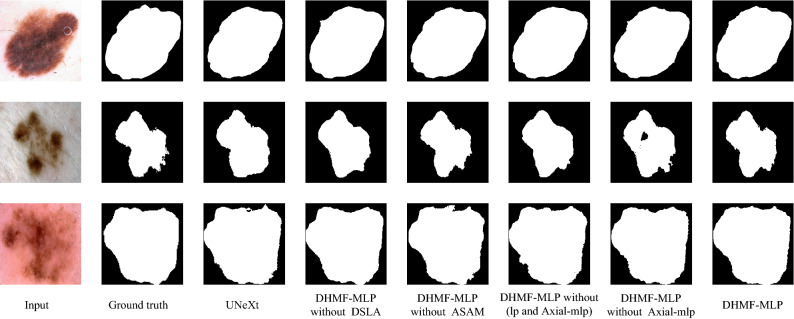
Ablation segmentation results of HMSF block on the ISIC 2018 dataset. From left to right: input, ground truth, UNeXt^22^, DHMF-MLP without DSLA, DHMF-MLP without ASAM, DHMF-MLP without (lp and Axial-mlp^21^), DHMF-MLP without Axial-mlp^21^, DHMF-MLP(Ours). (created by ‘Microsoft Office Visio 2013’ URL: https://www.microsoft.com/zh-cn/microsoft-365/previous-versions/microsoft-vision-2013).

To verify the individual contribution of each module in DHMF-MLP, we perform ablation experiments on three datasets and compare them with the baseline model (UNeXt^22^). (1) UNeXt^22^ framework; (2) DHMF-MLP without DSLA: our propose DHMF-MLP framework does not include DSLA module in its HMSF framework; (3) DHMF-MLP without ASAM: our propose DHMF-MLP framework with DSLA module without ASAM block; (4) DHMF-MLP without (lp and Axial-mlp^21^) Our proposed DHMF-MLP framework with DSLA modules does not have Axial-mlp^21^ blocks or learnable parameters. (5) DHMF-MLP without Axial-mlp^21^: our propose DHMF-MLP framework with DSLA module without Axial-mlp^21^ block; (6) DHMF-MLP: the DHMF-MLP framework is proposed by us. Tables 4, 5 and 6 show the quantitative analysis results of the ablation studies on the BUSI, ISIC 2018 and GlaS datasets, respectively. Figures 7, 8 and 9 show the qualitative analysis results of the ablation studies on the BUSI, ISIC 2018 and GlaS datasets, respectively.

**Table 4:** Ablation studies of the proposed blocks on the BUSI dataset.

Method	Params (in M)	IoU	Dice	SE	ACC	PPV	SP
UNeXt^22^	2.52	67.44	79.55	77.11	96.31	83.29	98.42
DHMF-MLP without DSLA	4.18	67.80	79.97	77.00	96.36	84.42	98.53
DHMF-MLP without ASAM	4.54	69.59	81.05	82.10	96.39	81.06	97.93
DHMF-MLP without (lp and Axial-mlp^21^)	4.18	68.54	80.66	81.19	96.26	80.70	97.93
DHMF-MLP without Axial-mlp^21^	4.18	69.29	81.21	79.51	96.43	84.05	98.28
DHMF-MLP	4.54	70.65	82.21	81.33	96.59	83.90	98.28

**Table 5:** Ablation studies of the proposed blocks on the ISIC 2018 dataset.

Method	Params (in M)	IoU	Dice	SE	ACC	PPV	SP
UNeXt^22^	2.52	82.55	90.28	89.08	95.87	92.14	97.91
DHMF-MLP without DSLA	4.18	82.88	90.52	89.90	95.57	91.60	97.73
DHMF-MLP without ASAM	4.54	83.21	90.68	91.01	95.98	90.91	97.45
DHMF-MLP without (lp and Axial-mlp^21^)	4.18	82.91	90.51	89.73	95.91	91.83	97.69
DHMF-MLP without Axial-mlp^21^	4.18	83.08	90.64	89.89	96.00	91.82	97.77
DHMF-MLP	4.54	83.46	90.84	89.69	96.18	92.54	98.08

**Table 6:** Ablation studies of the proposed blocks on the GlaS dataset.

Method	Params (in M)	IoU	Dice	SE	ACC	PPV	SP
UNeXt^22^	2.52	83.80	91.12	92.19	91.42	90.16	90.50
DHMF-MLP without DSLA	4.18	85.46	92.11	92.67	92.36	91.64	91.87
DHMF-MLP without ASAM	4.54	86.71	92.85	93.41	93.11	92.34	92.66
DHMF-MLP without (lp and Axial-mlp^21^)	4.18	86.03	92.44	92.51	92.81	92.40	92.92
DHMF-MLP without Axial-mlp^21^	4.18	86.42	92.66	92.87	92.94	92.50	92.77
DHMF-MLP	4.54	87.04	93.03	92.85	93.35	93.24	93.63

#### Quantitative result analysis

From the quantitative results in Tables 4, 5 and 6, our proposed DHMF-MLP without DSLA outperforms UNeXt^22^, verifying that multi-scale feature fusion can contribute to optimal segmentation results. The superiority of DHMF-MLP without Axial-mlp^21^ over DHMF-MLP without (lp and Axial-mlp^21^) indicates the importance of the learnable parameters. In addition, DHMF-MLP without Axial-mlp^21^ and DHMF-MLP without ASAM improve the segmentation metrics essentially without increasing the number of parameters and by increasing the number of parameters by less, respectively. We conclude the lightness of ASAM and Axial-mlp and the necessity of applying them to the feature fusion process. When they are all applied to the network, the IoU (%) of BUSI, ISIC 2018, and GlaS increases by 4.76%, 1.10%, and 3.87%, respectively.

#### Qualitative result analysis

Taking the first row of Fig. 8 as an example, DHMF-MLP without DSLA reduces redundant information through multi-scale feature aggregation, thus reducing over-segmentation. The addition of ASAM has positive influences on the adjustment of foreground and background information relationships compared to DHMF-MLP without DSLA, which is closer to the ground truth. The addition of Axial-mlp^21^ enhances the boundary segmentation effect, validating the module's ability to improve the network's ability to extract global contextual information. Compared with DHMF-MLP without (lp and Axial-mlp^21^), DHMF-MLP without Axial-mlp^21^ takes into account the difference of foreground and background information of different scale features and automatically adjusts itself by using the learnable parameters. From the segmentation results of the two columns in Fig. 8, the necessity of learnable parameters is proven.

As is vividly depicted in the third line of Fig. 9, DHMF-MLP without DSLA is much sharper in terms of edge profile compared to UNeXt^22^. That is, by fusing multi-scale features, rich semantic information is extracted, improving the segmentation effect. As shown in the second row of Fig. 9, DHMF-MLP without ASAM and DHMF-MLP without Axial-mlp^21^ achieve better boundary preservation results than DHMF-MLP without DSLA by utilizing global contextual information and adjusting for semantic conflicts that arise during the fusion process, respectively. The segmentation results from Fig. 9 show that the learnable parameters facilitate the adaptive adjustment of the semantic conflicts generated during the fusion process. The combination of the three of them significantly improves the segmentation effect and is closer to ground truth.

**Figure 10:**
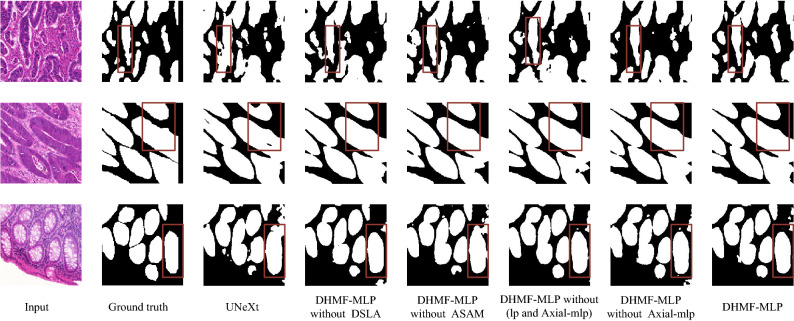
Ablation segmentation results of HMSF block on the GlaS dataset. From left to right: input, ground truth, UNeXt^22^, DHMF-MLP without DSLA, DHMF-MLP without ASAM, DHMF-MLP without (lp and Axial-mlp^21^), DHMF-MLP without Axial-mlp^21^, DHMF-MLP(Ours). (created by ‘Microsoft Office Visio 2013’ URL: https://www.microsoft.com/zh-cn/microsoft-365/previous-versions/microsoft-vision-2013).

According to the location of the red box in the second row shown in Fig. 10, DHMF-MLP without DSLA effectively reduces the under-segmentation of the lesion region compared to UNeXt^22^. This is due to the better identification of lesion regions through the interactive learning of low-level and high-level features. According to the red box position in the first row shown in Fig. 10, DHMF-MLP without Axial-MLP further utilizes learnable parameters to balance foreground and background information and reduce the adhesion between different lesion regions. DHMF-MLP without ASAM utilizes global contextual information to make the segmentation regions more complete. The feasibility of learnable parameters for adjusting semantic conflicts during fusion can be seen from the comparison results of DHMF-MLP without (lp and Axial-mlp^21^), DHMF-MLP without Axial-mlp^21^.

Through the above analysis, it can be seen that the quantitative results are consistent with the qualitative results. These experiments demonstrate the efficacy of our proposed method, which is exploited to extract rich multi-scale information for improving the accuracy of segmentation of small lesions and multi-lesion regions. Simultaneously, determine the feasibility of ASAM for adaptive learning of important features, as well as the necessity of Axial-mlp^21^ to retrieve global contextual information. When they are all applied to the network, as shown in the last column of the qualitative analysis results, they compensate for each other's flaws, resulting in significant improvements in the segmentation effect.

## Conclusion

We propose a new medical image segmentation framework called DHMF-MLP. HMSF is proposed as part of the encoder, which contains three functions. First, the accuracy of small lesion and multi-locus region segmentation is improved by fusing features from each stage of the encoder to obtain rich semantic information and reduce the deviation between features. Second, lightweight ASAM is constructed by applying learnable parameters to calculate feature weights based on the foreground and background information of the feature map to adjust the semantic conflicts arising from feature fusion. Third, Axial-mlp^21^, which is introduced to establish the global contextual information, fully compensates for the lack of global information at baseline and allows the fused feature information to be propagated so as to improve the overall performance of the network. Extensive experiments on three medical segmentation datasets have revealed that our proposed DHMF-MLP is competitive with current state-of-the-art methods. In the future, we will investigate the merits of the proposed DHMF-MLP on a wider range of datasets to improve the generalisation capability of the model.

## Data Availability

The BUSI, ISIC 2018 and GlaS datasets are openly available at: https://www.kaggle.com/aryashah2k/breast-ultrasound-images-dataset (accessed on 28 April 2022), https://challenge.isic-archive.com/data/ (accessed on 28 April 2022) and https://warwick.ac.uk/fac/cross_fac/tia/data/glascontest (accessed on 28 April 2022).
